# Rights to social determinants of flourishing? A paradigm for disability and public health research and policy

**DOI:** 10.1186/s12889-019-7334-8

**Published:** 2019-07-24

**Authors:** Maria Berghs, Karl Atkin, Chris Hatton, Carol Thomas

**Affiliations:** 1 Leicester School of Allied Health Sciences, De Montfort University, Leicester, England, UK; 2 Department of Health Sciences, University of York, York, England, UK; 3 Faculty of Health and Medicine, Furness College, Lancaster University, Lancaster, England, UK

**Keywords:** Public health, Disability, Intervention, Evidence, Policy, Flourishing

## Abstract

**Background:**

The term evidence based medicine was introduced in the early 1990s in clinical medicine to educate clinicians about how to assess the ‘credibility’ of research to ensure best treatments for their patients. The evidence based medicine paradigm has become more diffuse in times of austerity and randomised controlled designs are being used to address complex issues in public health and disability research. This research is not addressing inequalities in terms of disability nor how people can live well with disabilities.

**Main text:**

We argue that there are four ways that public health research needs to change if it wants to address inequalities linked to disability: 1) rethinking theoretical connections between public health and disability; 2) building ethics and equity into interventions through a human rights approach; 3) ensuring ethical inclusion through intersectionality; and 4) evaluating policy and other social impacts to ensure they capture diversity. We argue that these are key issues to building a social determinants of flourishing.

**Conclusions:**

We need to understand how disability might have an accumulative impact across the life course, as well as how to ensure equity for people living with disabilities. This means conceptualising a social determinants of flourishing where we evaluate how exactly randomised controlled trials and public health interventions, not only lead to greater equality but also ensure rights to health and wellbeing.

## Background

### Evidence based medicine, public health and policy-making

The term evidence based medicine (EBM) was introduced in the early 1990s in clinical medicine to educate clinicans about how to assess the ‘credibility’ of research to ensure best treatments for their patients [1]. British Centres for Evidence were established and it was taken up into clinical training, textbooks and practice. EBM was also espoused as new paradigm in 2001 by the Cochrane Collaboration, which publishes rigourous methodological information about clinical randomised controlled trials (RCTs) [2]. It was hoped that the EBM paradigm would ensure that clinical practices would become ‘scientfic’, in the sense of giving assurances of quality of scientific background and emprical research to inform clinical decisions, as well as evidencing value for money by ensuring clinicians did not pursue treatments that did not work [3].

Djulbegovic and Guyatt [1] argue that there are three epistemological principles for EBM: firstly that evidence has to be trustworthy, credibly determined and based on controlled clinical observatons; secondly that the ‘totality’ of evidence should inform the truth of decisions; and thirdly, that ‘clinical decision-making requires consideration of patients’ values and preferences’. In order to assess such principles, evidence hierarchies began to appear; with critical appraisals assuming that clinical RCTs provided more certainty than uncontrolled emprical studies, while systematic reviews (using evidence synthesis) were developed to measure the ‘totality’ of evidence. Parallel systems in the United States (US) have also evolved and numerous standards and guidelines have been developed to ensure better evaluations, as well as designs for RCTs. There has been a focus on what ‘works’ in terms of RCTs and then understanding ‘why’ in terms of their evaluation. Robust methodological guides are now emphasised in checklists, like the Consolidated Standards of Reporting Trials (CONSORT) [4]. In the United Kingdom (UK), the EBM paradigm has been espoused through the National Institute for Health and Care Excellence (NICE), who highlight the improvement of health and social care through evidence-based guidance. Implementation science has also been advocated by the National Health Service (NHS) to try and close the gap between research and practice, by evaluating ‘why’ interventions work.

In times of neoliberalism and austerity, it can seem as if the EBM framework is becoming more diffuse and applied to a much broader range of decisions [5], in line with an increasing emphasis being placed on evidence for budget allocations and justfications of how funding is being spent. Trial evidence has, therefore, become especially attractive to policy makers as it seems to offer potential solutions to complex, expensive and increasing politically contested dilemmas facing health care, thereby meeting ‘a longing that this rational discourse can in Rousseauian fashion locate and unmask our suffering’ [6]. This explains why the principles of EBM have spread, from being used to weigh up evidence for health interventions in clinical practices, to wider realms such as public health, social prescribing and even economic policy making. For example, the UK’s What Works Network was formed in 2014 as a government intiative that promotes the use of robust evidence to faciliate policy making and service delivery, in areas as diverse as health, policing and development aid [7]. This means, for instance, the UK’s Department for International Development (DFID) has to ensure that their policy teams can illustrate how decision making is evidence-based and this is inclusive of business decision-making [7]. While Greenhalgh et al. [3] and Wieringa et al. [5] have been critical of the misappropriation of the EBM brand, they also offer solutions, viewing EBM as a ‘situated practice’ in terms of links to how cultural values and norms influence ‘evidence’. This points to a trickier issue, in that while the policy focus is on what ‘works’, its evaluation and ‘why’ an intervention works becomes more complex in different global contexts.

In terms of public health, Victora et al. [8] too warn that application of EBM to research means that we have to become more critical and think beyound simplistic RCT designs. Similarly, Mays et al. [9] note that despite the fact that policy-makers are under increasing pressure to adopt evidence-based decisions, Cochrane style reviews of evidence alone will not be enough and different approaches might have to be evaluated. This indicates that issues with EBM are not only located within research but also policy making, affecting interventions and the evaluation of research evidence [10], for example in terms of implementation science, as well as its further funding. In this paper, we want to focus on the links between RCTs and public health to see if any evidence-based innovations are possible in terms of disability research.

RCTs are currently regarded as the gold standard for scientific evidence in public health policy, with gaps between research and policy uptake still viewed as problematic [11, 12]. We accept that RCTs in public health have a crucial part to play in ensuring the health of people who have disabilities across the life-course. Evidence from RCTs can lead to interventions that can tackle underlying causes of ill-health and reduce health inequalities with the potential to transform lives and ensure rights to independent living [13]. Yet, in a letter in *The Lancet,* van der Marck et al. [14] note that the biggest challenge that clinicians will face in the next twenty-five years, is patients presenting with multi-morbidities across the life-course for whom evidence informed guidelines will not work and may cause harm. They argue that there is a ‘fundamental mismatch’ between evidence being produced and what will be needed to tackle disabilities [14]. We argue that this mismatch in terms of disability is also present in public health, and linked to the kind of research that is being funded and its evaluation. This raises broader challenges in how evidence is presented and interpreted.

First, evidence synthesis reinforces the clinical basis of medicine, to the relative neglect of social-economic, cultural and environmental conditions [15], while simultaneously struggling to engage with the meaning patients and practitioners’ accord to an intervention [16]. Consequently, key contextual factors contributing to the broader social determinants of health - and the success or otherwise of an intervention - are underplayed [14]. Second, synthesis often assumes the idealised integrity of trial methodology, rather than offer a critical account of how the trial was conducted, designed and reported [17, 18]. Over 50 % of trial interventions, for example, are inadequately described and over 50 % of planned study outcomes not reported, with negative results rarely getting published [19]. Moreover, randomisation rarely provides representative samples, specifically failing to address diversity, in terms of age, sexuality, ethnicity or disability [13]. Third, a narrow focus on trial evidence has meant equally valuable forms of insight, such as those offered by epidemiology, are neglected [20]. Epidemiology has a particularly strong tradition of reflecting the experience of those traditionally neglected by trial evidence [21], and is also able to embrace the challenges posed by future of epigenetics [22]. Finally, the focus of trials, can obscure the role of political decision-making and bias in determining the evidence available or what evidence is regarded as significant and/or put into practice [23].

Despite decades of research advocating synergies between public health and disability research [24, 25], we know little about how disabled people and disability theory are integrated into public health RCTs. Previous studies have mostly recorded exclusions and non-recruitment of disabled population groups most affected by health inequalities, for example, people with intellectual disabilities [26–28]. We thus want to begin a critical theoretical and empirically informed discussion about the ways in which public health RCTs could better integrate disability. Furthermore, we argue that public health RCTs now need to take into account issues of policy impact, as well as health inequalities, which focus on ensuring not only that people can live well with disabilities but that they have a right to that health. This entails thinking more constructively, in practical terms, about how we are integrating human rights and social justice perspectives in inequalities research so we guarantee social flourishing with disabilities. Our approach is consistent - at least in spirt - to the intent of Doll & Bradford-Hill [29], who in establishing the scientific basis of current trial methodology, were suspicious of naïve descriptive empiricism, particularly when it was at the expense of more theoretically informed enquiry. We argue for a novel social determinants of flourishing, which is more consistent with how people experience disability, within an evidence-based framework that places an emphasis on RCTs and their evaluation. There are four ways in which this can be achieved by: 1) Rethinking theoretical connections between public health and disability; 2) Building ethics and equity into interventions through human rights; 3) Ensuring ethical inclusion through intersectionality; and 4) Evaluating policy and other social impacts. We explain each in turn.

### Social flourishing with disabilities

#### Rethinking theoretical connections between public health and disability

In a commissioned National Institute for Health Research - Public Health Research (NIHR-PHR) study on the implications of disability for public health RCTs, we did a global scoping review of 30 specific public health systematic reviews as well as 30 generic systematic reviews of RCTs found in the Cochrane database. We evaluated these reviews through a disability rights framework and we found that there had been limited public health engagement with the theories and models of both public health and disability [13]. Theory based RCTs and their efficacy have received increasing public health attention in terms of explaining the outcomes of interventions [30, 31]; theoretical disconnects of RCTs to disability theories and models much less so. These theoretical disconnections between the public health paradigms that are being espoused in RCTs and actual research involving disability should get more critical attention [32]. If we examine the need for theoretically informed RCTs, we note that there has to be a greater theoretical public health engagement *with* personal values and ethical norms of disabled people, as well as theory found in disability studies literature.

We argue that disability theory should be central to the design of RCTs undertaken by public health researchers, as well as their critical evaluations and thus incorporated in systematic reviews. Da Silva et al. [33] argue that, for increasingly complex prospective interventions, more rigorous theoretical approaches to intervention design are needed. However, to understand why interventions are working either upstream or downstream also requires critical theoretical evaluation of the epistemological and ontological foundations of public health and disability RCTs. Why particular RCT designs being chosen over others, and do they work in practical terms? Are they cost-effective short and long term; and what frameworks are being used to make those judgements about cost-effectiveness? Are the best public health and disability RCT designs necessarily complex? Why and when do explanatory or pragmatic designs work?

To answer these questions, some authors advocate using realist approaches when evaluating public health RCTs [34, 35], and some even when designing interventions in general [36]. This could connect well with the theoretical and methodological basis of disability models and with more holistic, complex and ecological understandings of public health [13, 37]. Such approaches could also offer a critical commentary on what questions get asked in the first place, alongside how studies are conducted and findings interpreted. Another way in which to evaluate and rethink the way in which we design public health and disability RCTs, would be to use human rights as a bridge.

#### Building equity into RCT design and evaluation through human rights

Human rights frameworks or approaches often use ‘persons first’ definitions and aim to establish legal, political, cultural, social and economic rights for all people [13]. Human rights theories can provide a unifying framework or bridge between public health, and disability theories and models, to ensure equity [38]. Despite the potential of human rights frameworks, we found that they have been somewhat neglected in RCTs in terms of design and evaluation [13]. This is despite a general trend among institutions, such as the World Health Organization (WHO) and United Nations (UN), in adopting human rights indicators, frameworks, measurements of health capabilities and health equity monitoring to evaluate interventions in general.

Rights-based approaches are also increasingly being used in terms of RCT design as well as evaluations of public health and disability interventions [27, 39]. Human rights and equality frameworks are advocated in terms of social protection and to safeguard entitlements and rights to health. We also found that there was not only a political and social acceptance of human rights frameworks amongst people with disabilities in the UK but also advocacy for greater enforcement in all areas of life, including public health research [40]. While human rights frameworks, and in particular the United Nations Convention on the Rights of Persons with Disabilities (CRPD) [41] is often mentioned in research, how to operationalise such rights to be used in design and evaluative tools in RCTs and general public health interventions has been under theorised. Notwithstanding the fact that there is a greater need to think about not only how to gather evidence of public health impact on health inequalities [42] but now also assurances of rights of people affected [40].

Human rights frameworks are also in keeping with critical disability theories that destabilise the norms of rational choice theory and emphasise the social causes of health inequalities rather than ‘checking’ the health effects of RCTs. Furthermore, this is consistent with public health paradigms that advocate complexity and innovation, and as such could be easily taken up into guidelines that evaluate RCT designs and interventions. The CRPD in particular, encapsulates that health is about more than medical access and disability results ‘from the interaction between persons with impairments and attitudinal and environmental barriers that hinders their full and effective participation in society on an equal basis with others’ [41].

#### Ensuring ethical inclusion through intersectionality

In terms of equity, Schulz et al. [4] note that a weakness in checklists like CONSORT is that they do not record that information. Yet, equity can be easily integrated and considered in consultation, design and impact of RCTs. This is especially important in public health, when RCTs are designed in response to health inequalities amongst marginalised population groups, for example, disabled people. To overcome this difficulty, we introduce the idea of design and evaluation of equity within interventions through the use of disability theory and human rights frameworks. Research indicates that social justice in terms of impact of RCTs, is now also becoming linked to greater inclusion for disabled people [40]. Inclusion would also involve intersectionality of disability to age, sexuality, ethnicity, gender, socio-economic status and co- or multi-morbidities. However, we think intersectionality has to be broader than this to ensure ethical inclusion.

A next step, in terms of inclusion and an intersectional approach, would be to evaluate equity downstream, in terms of impact of an RCT not just on policy but in terms of developing an understanding of individual, socio-political, economic and environmental impacts on disability, such as via the social determinants of health [43]. Humphreys and Piot [44] have argued that scientific evidence alone is not a sufficient basis for health policy. We would argue that political legitimation of policy weakens without a link to empirically and theoretically robust science.

Liverani et al. [45] note that there has been ‘limited explicit engagement with relevant theories in the literature on evidence-informed health’ and they argue more research on bias and policy uptake of evidence-based research is needed. Recognising the broader determinants (individual, social, political and economic) of health could facilitate more inclusive research practices and allow researchers to locate an individual’s intersectional and epi-genetic experiences within their social environment [42]. Thus equity upstream and downstream of RCTs would have to be assured in terms of inclusion and intersectionality.

#### Evaluating policy and other social impacts

The uptake of evidence of RCTs into generalised public health interventions and their evaluations is the responsibility of public health policy makers, commissioners or providers. This involves political decision making. Similarly, the funding priorities and research that is being commissioned is decided by political requirements, understanding of health priorities and policy trends. RCTs are viewed as a gold standard in terms of influence on policy but within public health and disability research it would be useful to ensure a means of ‘social accountability’ through evaluation of improvement or attainment of disability rights or acquisition of rights by disabled people during and after a RCT [46]. Within public health research, and implementation science in particular, there have been many suggestions to develop conceptual frameworks to evaluate equity in RCT designs [47] but there is no main standard or guideline that is being used. Likewise, within policy-making there is no standard guideline for assessing the social impacts of a public health intervention and no real links to equity similar to, for instance, the National Health Services (NHS) Equality Analysis and Impact Assessments. We argue that impacts and assessments in both RCT research design and intervention studies have to move beyond equity and inequalities, towards understanding if people’s rights to social determinants of flourishing are being respected.

This means that the focus would also shift from influencing policy to understanding social policy impacts, for example, on rights entitlements or people with protected characteristics. This is about more than ensuring equity or assessing inequalities. We argue that RCTs would have to integrate evaluations of equity and translation of equity indicators, or measures that aligned with public health and disability theory, for example, and this would translate into evaluating how ‘enabling’ a public health RCT was to both the short and long-term sustainability of wellbeing of people with disabilities. Another means of understanding public health wellbeing or equity would be to examine the capabilities that people had before and after a public health intervention and if they were able to sustain, live well or ‘flourish’ with impairment(s) [48].

In terms of conceptualising what a more equitable public health and disability paradigm shift would include, we contend that an evaluation of how people flourish or thrive is consistent with both public health and disability theories and models. Rather than conceptualising disability in terms of burden, cost, reduced functioning or viewing aging as a problem to public health, we feel that we could advocate a more holistic understanding by focusing on social and environmental impacts. Such an understanding would take a different ontological and epistemological approach to equity in public health, in terms of shift to a social measurement of not only right to health or capability for health but also a conceptualisation of how RCTs and interventions aid people with disability, chronic illness and impairment to live well across the life-course. The understanding of ‘flourish’ is different from those developed in terms of ‘capabilities’ because it encompasses elements of distinction, which are connected to social and political empowerment.

This would encompass a finer attunement to the impact of ill health as well as disabling experiences and environments. So, an evaluation could be translated in terms of measures of how and if public health interventions had an impact on social status or standing, environmental accessibility or political emancipation and how sustainable this proved to be [49]. Most measures and indicators that have been developed either work in a specific disability model [48] or have not engaged public health *alongside* disability. We are arguing that evaluations within RCTs examining equity or assessing outcomes of interventions should focus on social determinants of flourishing for people with disability and impairment across the life-course.

## Conclusion

Disability is a continuous process and everyone is likely to be affected by ill health, impairment and disability across the life-course as they age and people with disabilities are living longer. Yet, the inequalities affecting disabled people living in poverty, disabled children, and those with intellectual and complex disabilities mean we have more and better work to do. There are now over 10 million people who face ‘limitations in daily activities’ in the UK and disability is found more in areas of greater social disadvantage [50]. Epidemiologically, disability is also linked to illness and disease across the life course with extra needs corresponding to impairment and co-morbid conditions [51]. From a public health perspective, disabled people are disadvantaged in all aspects of their life: from socio-economic environments in which they live to lack of access to quality housing; education; transport; and health and social care services - this has a cumulative affect across the life-course [52].

Research has found reduced life expectancy among people with intellectual disabilities and those with mental health conditions. For instance, Heslop et al. [53], in their confidential inquiry, reported that men with intellectual disabilities die 13 years earlier and females 20 years earlier than the general population. The social determinants of mental health of people with intellectual disabilities, has also been correlated with ‘poorer living conditions’ rather than ‘impairment per se’ [54]. Evidence found higher rates of hospitalisations [55] and increases in mortality rates [56] but also that public health interventions could be focused to better aid this under-served population group [57].

We need to understand more about how disability might have an accumulative impact across the life course, as well as how epidemological factors like epigenetics play a role in understanding equity. We argue that an evidence-base focused on how to *flourish* with disability would provide those answers. RCTs and interventions can become more ethical and empirically robust by reconceptualising inclusion of public health *with* disability theory as well as include disabled people in making those changes, enabling everyone to live well and flourish. This means conceptualising a social determinants of flourishing where we evaluate how exactly RCTs and public health interventions, not only lead to greater equality but also ensure rights to health and wellbeing.

## Data Availability

The data and materials are available online and this has been cited in the text. See Berghs et al. 2016.

## Abbreviations

EBM: Evidence Based Medicine
NHS: National Health Service
NICE: National Institute for Health and Care Excellence
RCT: Randomised Controlled Trial
